# Severe Cases of Pandemic (H1N1) 2009 in Children, Germany

**DOI:** 10.3201/eid1702.101090

**Published:** 2011-02

**Authors:** Mathias Altmann, Lena Fiebig, Jana Soyka, Rüdiger von Kries, Manuel Dehnert, Walter Haas

**Affiliations:** Author affiliations: Robert Koch Institute, Berlin, Germany (M. Altmann, L. Fiebig, J. Soyka, M. Dehnert, W. Haas);; Ludwig-Maximilians-Universität, Munich, Germany (R. von Kries)

**Keywords:** Pandemic (H1N1) 2009, influenza, viruses, infants, children, critical illness, risk factors, Germany, research

## Abstract

In a hospital-based observational study in Germany, we investigated children admitted to pediatric intensive care units and deaths caused by confirmed pandemic (H1N1) 2009 to identify risk factors and outcomes in critically ill children. Ninety-three children were eligible for our study, including 9 with hospital-acquired infections. Seventy-five percent had underlying chronic medical conditions; neurodevelopmental disorders were most prevalent (57%). The proportion of patients having >1 risk factor increased with age in years (odds ratio 1.21, p = 0.007). Of 15 deaths, 11 occurred in a pediatric intensive care unit (case-fatality rate 12%, 95% confidence interval 6%–21%). Only 9% of the children had been vaccinated against pandemic (H1N1) 2009; all survived. Our results stress the role of underlying risk factors, especially neurodevelopmental disorders, and the need for improving preventive measures to reduce severe disease and adverse outcomes of pandemic (H1N1) 2009 in children.

The novel strain of influenza known as pandemic (H1N1) 2009 virus that originated in Mexico and the United States resulted in the first pandemic of the 21st century. Cases were observed in 214 countries, and 18,097 laboratory-confirmed deaths caused by this virus have been reported (*1*). In Germany, where the first cases were confirmed on April 29, 2009, the number of reported cases was 226,158 (including 255 deaths) as of May 18, 2010 (*2*).

Children were particularly affected by pandemic (H1N1) 2009. This finding is evident in the age distribution of patients, which is skewed toward younger age groups, and in high hospitalization rates for children identified in many settings worldwide (*3**–**5*). Severity has been mostly assessed in terms of admission to intensive care units (ICUs) and case-fatality rates. In a cohort study in Australia and New Zealand, the highest age-specific incidence rate for ICU admission was for children <1 year of age (*6*). Observational studies in ICU settings in the early pandemic phase in Mexico (*7*) and Canada (*8*) highlighted high rates of adolescents among critically ill patients.

Studies conducted in pediatric ICU (PICU) settings originate predominantly from the Americas (*9**–**11*). In Europe, Lister et al. summarized experiences from 4 ICUs in the United Kingdom and identified 13 critically ill children with pandemic (H1N1) 2009 during June–July 2009 (*12*). These studies and national surveillance systems contributed to a better understanding of determinants and risk factors for severe disease in children. However, information from countries in Europe about severe cases of pandemic influenza (H1N1) 2009 in children who are particularly vulnerable is still limited (*12**,**13*). To obtain information on risk factors, course of disease, and outcome of critically ill children with pandemic (H1N1) 2009, we prospectively performed a nationwide observational study covering the fall wave of pandemic (H1N1) 2009 in Germany.

## Methods

### Study Design

We investigated cases of critically ill children with confirmed pandemic (H1N1) 2009 in pediatric hospitals in Germany. The 375 study sites participating in the established nationwide active surveillance network Survey Center for Rare Pediatric Diseases in Germany (ESPED) comprise all pediatric hospitals in Germany.

The study included pediatric cases of pandemic (H1N1) 2009 reported during August 2009–April 30, 2010. Cases were defined as illness in patients <15 years of age who had a laboratory-confirmed infection with pandemic (H1N1) 2009 virus (determined by PCR, virus isolation, or antigen detection) and were admitted to a PICU or died.

### Data Collection

**Figure 1:**
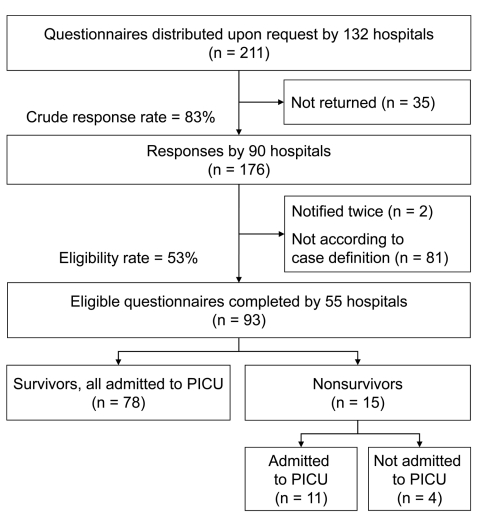
Overview of study participation and participant groups among children with severe pandemic (H1N1) 2009, Germany, 2009–2010. PICU, pediatric intensive care unit.

A structured questionnaire, adapted from an earlier study on seasonal influenza by Liese et al. (*14*), was distributed to the hospitals that reported cases, completed by the treating physician, and collected by the ESPED study center. Monthly reporting was requested even if no cases were identified. Up to 3 reminders were sent if questionnaires were not returned. To take into account the reporting delay, we included reports received by the study center until the end of April 2010. Of 211 distributed questionnaires requested by 132 hospitals, 176 (83%) were returned to the study center (Figure 1). After excluding 2 patients with cases who had been notified twice and 81 questionnaires from persons who did not fulfill the case definition, there remained 93 (53%) eligible questionnaires from 55 hospitals. Data were double entered into an electronic database by using EpiData software (EpiData Association, Odense, Denmark). Individual datasets were inspected for missing information, plausibility, and data entry errors. The contact persons of the participating hospitals were notified up to 2 times when data were incomplete in the questionnaire form.

The structured questionnaire included patient information; data on the hospital stay; clinical signs and symptoms of illness; clinical and laboratory diagnosis; specific treatments; underlying chronic medical conditions (chronic respiratory diseases, cardiac diseases, immunodeficiency and neurodevelopmental disorders, including developmental delay, cerebral palsy, epilepsy, and other cognitive disorders); status of influenza vaccination; and complications of the disease. Answer categories were predetermined, but other diagnoses and concurrent conditions could additionally be specified by the respondents as free text.

### Statistical Analysis

Descriptive statistics comprised the calculation of median and interquartile ranges (IQRs) for continuous variables and absolute numbers and proportions (together with 95% binomial exact confidence intervals [CIs] where appropriate) for categorical variables. For the calculation of inpatient periods, patients were excluded if they had acquired pandemic (H1N1) 2009 while hospitalized. Comparative analyses were performed on the basis of the Wilcoxon rank-sum test for continuous variables and Fisher exact test for categorical variables only for patients admitted to a PICU. Odds ratios (ORs) and 95% CIs were calculated. Logistic regression was performed for continuous independent variables. All reported p values were 2-sided, and p<0.05 was considered significant. Statistical analyses were performed by using Stata 11.0 (StataCorp LP, College Station, TX, USA).

### Data Protection and Ethical Clearance

Adherence to national data protection laws was approved by the Federal Commissioner for Data Protection and Freedom of Information of Germany. Ethical approval was granted by the Ethics Committee, Charité, Universitätsmedizin, Berlin, Germany.

## Results

### Characteristics of Study Population

**Figure 2:**
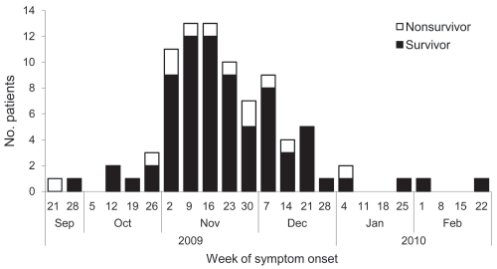
Date of symptom onset for 86 children with severe pandemic (H1N1) 2009, Germany, September 21, 2009–February 22, 2010. Only children with available information are included.

**Figure 3:**
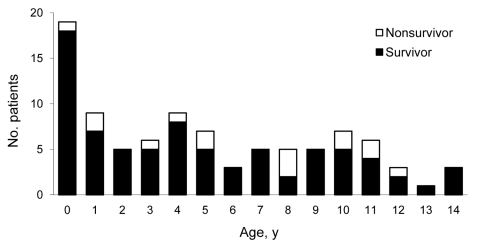
Age distribution of 93 children with severe pandemic (H1N1) 2009, Germany, 2009–2010.

During the study period, we included 93 critically ill children with confirmed pandemic (H1N1) 2009. Their dates of disease onset were September 21, 2009–February 23, 2010; a peak in November 2009 included 58% of the cases (Figure 2). Sixty percent of the patients were boys. The age distribution is shown in Figure 3. Median age was 4.5 years (IQR 1.3–9.3 years), 19 (20%) were <1 year of age, and 16 (17%) children were <6 months of age. Seventy-eight patients survived and 15 died. Among those who died, 4 patients were not admitted to a PICU (Figure 1). Nine patients, of whom 1 died, had acquired pandemic (H1N1) 2009 while hospitalized. The PICU cohort comprised 89 patients with a case-fatality rate of 12% (95% CI 6%–21%). The 89 reported patients correspond to an incidence rate for severe PICU-admitted cases of 27.8 cases/million children in infants <1 year of age and 8.0 cases/million children in children <15 years of age (all children of the same age group). No difference was found in the age distribution between survivors and those who died.

### Underlying Chronic Medical Conditions and Vaccination Status

**Figure 4:**
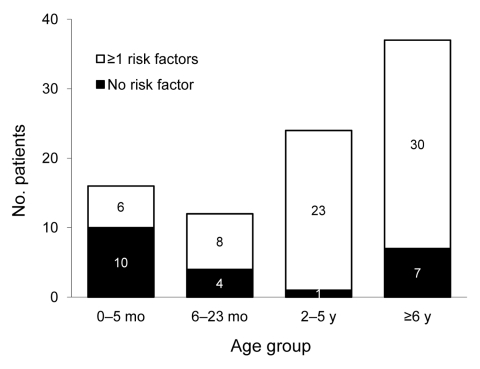
Age group distribution of 89 children with severe pandemic (H1N1) 2009, by number of underlying chronic medical conditions (risk factors), Germany, 2009–2010. Only children with available information are listed. Risk factors are chronic respiratory diseases, cardiac diseases, immunodeficiency, and neurodevelopmental disorders.

Seventy-five percent (67/89 with available information) of the patients had ≥1 underlying chronic medical condition known as a risk factor for seasonal influenza. The age distribution by presence or absence of ≥1 underlying chronic medical condition is shown in Figure 4. The proportion of patients having ≥1 risk factor increased with age in years (OR 1.2, 95% CI 1.1–1.4; p = 0.007). Neurodevelopmental disorders were most frequently reported (57% of the cases), followed by chronic respiratory diseases (38%), immunodeficiency (16%), and cardiac diseases (13%) (Table 1). Neurodevelopmental disorders were associated with a chronic respiratory disease in 60% (25/42) of the cases and were present in 79% (11/14) of those who died. Among the 53 children >6 months of age for whom information was available, 5 patients (9%) had been vaccinated against pandemic (H1N1) 2009; all of them survived.

**Table 1:** Underlying chronic medical conditions and vaccination status for children with severe pandemic (H1N1) 2009, Germany, 2009–2010*

Characteristic	Total	Nonsurvivors not in PICU	Admitted to PICU				
			Survivors	Nonsurvivors	Subtotal	p value	OR (95% CI)
Underlying chronic medical conditions							
Any	67/89 (75)	4/4 (100)	56/76 (74)	7/9 (78)	63/85 (74)	1	1.3 (0.2–13.3)
Neurodevelopmental disorders	51/89 (57)	4/4 (100)	40/75 (53)	7/10 (70)	47/85 (55)	0.501	2.0 (0.4–13.1)
Respiratory disease	31/82 (38)	2/3 (67)	25/70 (36)	4/9 (44)	29/79 (37)	0.718	1.4 (0.3–7.3)
Immunodeficiency	13/80 (16)	1/4 (25)	12/67 (18)	0/9 (0)	12/76 (16)	0.339	0.0 (0.0–2.1)
Cardiac disease	11/84 (13)	0/4 (0)	9/70 (13)	2/10 (20)	11/80 (14)	0.621	1.7 (0.2–10.6)
Vaccination status in patients >6 mo of age							
Any influenza	9/56 (16)	0/4 (0)	9/48 (19)	0/4 (0)	9/52 (17)	1	0.0 (0.0–4.7)
Pandemic (H1N1) 2009	5/53 (9)	0/4 (0)	5/45 (11)	0/4 (0)	5/49 (10)	1	0.0 (0.0–9.2)

*Values are no. positive/no with available information (%) except as indicated. PICU, pediatric intensive care unit; OR, odds ratio; CI, confidence interval. ORs were calculated only among patients admitted to the PICU.

### Clinical Manifestations

Pneumonia was the most frequent clinical diagnosis. It was documented in 70 (75%) of 93 patients and was the only diagnosis for 37% of them. The second most frequent diagnosis was acute respiratory distress syndrome (ARDS) in 22 (24%) of 93 patients. This diagnosis was only reported for patients admitted to a PICU and was associated with death (OR 7.4, 95% CI, 1.6–37.8; p = 0.004). Six patients had a diagnosis of encephalopathy and 2 had a diagnosis of myocarditis (Table 2).

**Table 2:** Clinical diagnosis for children with severe pandemic (H1N1) 2009, Germany, 2009–2010*

Clinical diagnosis	Total†	Nonsurvivors not in PICU†	Admitted to PICU				
			Survivors†	Nonsurvivors†	Subtotal†	p value	OR (95% CI)
Pneumonia	70/93 (75)	4/4 (100)	59/78 (76)	7/11 (64)	66/89 (74)	0.465	0.6 (0.1–2.9)
ARDS	22/93 (24)	0/4 (0)	15/78 (19)	7/11 (64)	22/89 (25)	0.004	7.4 (1.6–37.8)
Secondary pneumonia	15/93 (16)	0/4 (0)	13/78 (17)	2/11 (18)	15/89 (17)	1	1.1 (0.1–6.3)
Bronchitis/bronchiolitis	12/93 (13)	1/4 (25)	10/78 (13)	1/11 (9)	11/89 (12)	1	0.7 (0.0–5.8)
Encephalopathy	6/93 (6)	0/4 (0)	5/78 (6)	1/11 (9)	6/89 (7)	0.558	1.5 (0.0–15.1)
Sepsis	6/93 (6)	1/4 (25)	5/78 (6)	0/11 (0)	5/89 (6)	1	0.0 (0.0–5.6)
Status asthmaticus	2/93 (2)	0/4 (0)	2/78 (3)	0/11 (0)	2/89 (2)	1	0.0 (0.0–14.5)
Febrile seizure	2/93 (2)	0/4 (0)	2/78 (3)	0/11 (0)	2/89 (2)	1	0.0 (0.0–14.5)
Myocarditis	2/93 (2)	0/4 (0)	1/78 (1)	1/11 (9)	2/89 (2)	0.233	7.7 (0.1–611.9)
Other diagnosis‡	26/93 (28)	0/4 (0)	21/78 (27)	5/11 (45)	26/89 (29)	0.287	2.3 (0.5–9.9)

*Values are no. positive/no. with available information (%) except as indicated. PICU, pediatric intensive care unit; OR, odds ratio; CI, confidence interval; ARDS, acute respiratory distress syndrome. ORs were calculated only among the cases admitted to the PICU. †Children may have had >1 diagnosis. ‡Acute exacerbation of a chronic disease or new diagnosis.

### Hospital Course and Treatment

The median duration from symptom onset to hospital admission was 2 days (IQR 1–5 days), and the median duration from hospitalization to PICU admission was 0 days (IQR 0–1 days) for all patients admitted to the PICU. Both of these periods were not different between surviving patients and those who died in the PICU. Among those who died who were treated in the PICU, the median time from symptom onset to death was 8 days (IQR 3–12 days), and the median length of stay in the PICU was 2 days (IQR 0–8 days) (Table 3).

**Table 3:** Clinical course for children with severe pandemic (H1N1) 2009, Germany, 2009–2010*

Characteristic	Total	Nonsurvivors not in PICU	Admitted to PICU			
			Survivors	Nonsurvivors	Subtotal	p value
Time course of illness, d						
Symptom to hospital admission	77, 2 (1–5)	3, 1 (1–2)	65, 2 (1–5)	9, 1 (1–3)	74, 2 (1–5)	0.700
Hospitalization to PICU admission	74, 0 (0–1)	NA	65, 0 (0–1)	9, 1 (0–3)	74, 0 (0–1)	0.236
PICU length of stay	80, 8 (3–17)	NA	69, 9 (3–18)	11, 2 (0–8)	80, 8 (3–17)	NC
Hospital length of stay	83, 14 (5–23)	3, 5 (3–12)	69, 16 (7–25)	11, 3 (2–12)	80, 14.5 (5.5–23.5)	NC
Symptom onset to outcome†	85, 16 (8–26)	4, 5.5 (5–9.5)	72, 18.5 (10.5–29.5)	9, 8 (3–12)	81, 17 (8–27)	NC
Time to treatment, d						
Symptom onset to oseltamivir treatment	45, 4 (1–7)	1, 4‡	39, 4 (1–7)	5, 4 (2–8)	44, 4 (1–7)	0.551

*Values given are total no. with available information, median (IQR) ,except as indicated. PICU, pediatric intensive care unit; IQR, interquartile range; NA, not applicable (not admitted to PICU); NC, not compared because of different outcomes (release for survivors and death for nonsurvivors). †Including patients with hospital-acquired pandemic (H1N1) 2009 infection. ‡Only 1 observation.

Among patients admitted to the PICU, oseltamivir was administered to 61% (51/84) of the patients; there was no difference in its use between survivors and those who died (Table 4). Median time from symptom onset to antiviral treatment for both groups was 4 days (IQR 1–7 days for survivors and 2–8 days for those who died) (Table 3). Other treatments in the PICU included catecholamines (28/81 PICU patients, 35%) and mechanical ventilation (56/86, 65%). Both of these treatments were administered more often to those who died (p = 0.007 and p = 0.007, respectively).

**Table 4:** Treatment administered to children with severe pandemic (H1N1) 2009, Germany, 2009–2010*

Treatment	Total	Nonsurvivors not in PICU	Admitted to PICU

Survivors	Nonsurvivors	Subtotal	p value	OR (95% CI)
Oseltamivir	53/88 (60)	2/4 (50)	44/74 (59)	7/10 (70)	51/84 (61)	0.733	1.6 (0.3–10.2)
Antimicrobial drug	80/91 (88)	4/4 (100)	67/77 (87)	9/10 (90)	76/87 (87)	1	1.3 (0.2–64.7)
Catecholamine	28/85 (33)	0/4 (0)	20/70 (29)	8/11 (73)	28/81 (35)	0.007	6.7 (1.4–41.8)
Mechanical ventilation	56/90 (62)	0/4 (0)	45/75 (60)	11/11 (100)	56/86 (65)	0.007	NA (1.8–NA)

*Values are no. positive/no with available information (%) except as indicated. PICU, pediatric intensive care unit; OR, odds ratio; CI, confidence interval, NA, not applicable.

## Discussion

During the peak phase of the pandemic, active surveillance in pediatric hospitals identified 93 severe cases of pandemic (H1N1) 2009 in children with available information on prior medical history, course of disease, and outcome. When we compared absolute numbers, deaths of children caused by pandemic (H1N1) 2009 were reported 23× more frequently in our study than in a prospective study on seasonal influenza (*14*). In this study, which used an analogous case definition and the same hospital network, the deaths of only 2 patients were reported for 3 influenza seasons (2005–06, 2006–07, and 2007–08 seasons) in Germany. Similarly, in the United States, more deaths in children caused by pandemic (H1N1) 2009 were reported than for each of the 3 previous influenza seasons (*15*).

The higher number of reported deaths caused by pandemic (H1N1) 2009 might be partially explained by the high level of suspicion among physicians during the pandemic, which resulted in more frequent testing and diagnosis of influenza. This hypothesis is supported by a prospective study for seasonal influenza in the United States, in which only 43% of children admitted to a PICU with laboratory-confirmed influenza were independently given a diagnosis of influenza by the treating physician (*16*).

Our study indicated a PICU case-fatality rate of 12%, which is consistent with results from a study in Canada, which reported a case-fatality rate of 7% among 57 case-patients admitted to a PICU (*11*). However, case-fatality rates for children with pandemic (H1N1) 2009 vary considerably across study sites, as shown in 2 other studies in PICU settings. In a cohort of 147 children in Argentina, a case fatality rate of 39% was reported (*9*), which was similar to a case-fatality rate of 38% in a cohort of 13 patients in the United Kingdom (*12*). Both studies reported a higher case-fatality rate than for seasonal influenza. Differences in health care organization, including PICU admission criteria, age structure of the cohorts, and selection of study sites may partly explain the different findings.

The incidence rate for severe cases in PICU-admitted patients <15 years of age was 8.0 cases/million children, which was 5× times as high as the cumulative incidence over the 3 previous influenza seasons in the same population group reported by Liese et al. (1.7 cases/million children in the same age group) (*14*). This finding is consistent with studies from Australia and New Zealand, which showed the highest age-specific incidence in this age group (*6*).

Children <1 year of age represented 20% of our cohort, and thus a higher proportion than in the cohort investigated in the Netherlands (15%) (*13*). Special awareness is clearly needed for diagnosing influenza in infants because of the variable clinical manifestations in this age group. This awareness might be particularly relevant in low-resource settings that have limited virologic diagnostic capacities.

In our study, ARDS and pneumonia were the most frequent diagnoses among those who died. ARDS was the only diagnosis strongly associated with a fatal outcome (PICU case-fatality rate 32%). In Argentina, 80% of the children in a PICU had ARDS, and this condition was also associated with death (*9*). The frequency of other complications, which included 6 cases of encephalopathy and 2 cases of myocarditis did not differ between survivors and those who died.

Nine of 93 children in our study had acquired pandemic (H1N1) 2009 while hospitalized. The risk for nosocomial transmission of pandemic (H1N1) 2009 has also been documented in other studies (*17**,**18*). In both of these reports, pandemic (H1N1) 2009 was likely transmitted by health care workers. Additionally, children with underlying chronic medical conditions might have a higher risk for being hospitalized and therefore are particularly exposed to the risk for nosocomial infection. As reported for seasonal influenza (*19**,**20*), this result stresses the need for appropriate preventive strategies in hospital settings, such as early use of diagnostic tests and vaccination of health care workers who are involved in the care of patients with risk factors for severe disease.

We observed that patients who died had a median time in the hospital of 3 days, including 2 days in a PICU. Death occurred despite maximum intensive care therapy, as demonstrated by the higher rate of catecholamine treatment and mechanical ventilation among those who died. This observed rapid course of fatal disease despite intensive care, which was also reported in the United Kingdom (*12*), underlines the need for prevention.

The proportion of patients having >1 underlying chronic medical condition was high (75% overall) and increased with age. Our findings are consistent with those from a case series of 235 hospitalized children with pandemic (H1N1) 2009 in Canada (median age 4.8 years, range 0–16 years). A total of 60% of the patients in this study had >1 underlying chronic medical conditions (33% were children <2 years of age and 72% were older children) (*21*). Neurodevelopmental disorders were reported for more than half of the children and in more than three fourths of those who died. These results are consistent with the results from other PICU-setting studies in which neurodevelopmental disorders were the first or second most prevalent risk factor (*9**–**12*). According to the surveillance system for pediatric deaths associated with pandemic (H1N1) 2009 in the United States, 92% of the children with high-risk medical conditions had neurodevelopmental disorders (*22*).

In our study, only 5 children had been vaccinated against pandemic (H1N1) 2009. Their vaccination dates were not given, and it remains unclear whether the interval was sufficient to acquire immune protection. A considerable proportion of the patients with investigated cases could not benefit from immunization because the pandemic (H1N1) 2009 vaccine was not publicly available in Germany until after November 2, 2009, and 17% of all children in this study were <6 months of age. In Germany, neurodevelopmental disorders had not been explicitly included in the chronic medical conditions in the vaccination recommendations for seasonal influenza (*23*) and were only specified in recommendations for pandemic (H1N1) 2009 vaccine (*24*). In contrast, in the United States, neurodevelopmental disorders had already been recognized as a risk factor for seasonal influenza in 2005 (*25*).

Recent reports on adults and children with pandemic (H1N1) 2009 suggested that oseltamivir therapy benefitted patients with severe cases. Early treatment within 2 days after symptom onset was statistically associated with a lower risk for ICU admission and death in hospitalized pandemic (H1N1) 2009 patients (n = 272; median age 21 years) than with later treatment (*26*). In our study, the median time to oseltamivir treatment was 4 days and did not differ between survivors and those who died. Therefore, our study might not have been able to detect the benefit of this treatment. Nevertheless, this finding should be viewed with caution because our study was not designed to evaluate the effectiveness of oseltamivir for treatment of children with pandemic (H1N1) 2009. However, 1 ICU-setting study (n = 58; median age 44 years) suggested a benefit for patients who were treated with oseltamivir >48 hours after illness onset (*7*).

The representativeness of our study was assessed by comparing our data with those from the national databases. First, the timeline of our cases was compared with the Praxis Index, which derives from the syndromic surveillance system of the national working group on influenza and accounts for all notifications of influenza-like illness cases in Germany. The Praxis Index curve and the epidemiologic curve of patients investigated in our study show similar shapes. Second, of the 15 identified deaths in our study, 14 could be matched with the 29 deaths in children <15 years of age reported in the National Surveillance System. This difference might be explained by the fact that only children admitted to pediatric hospitals were captured in our study. Because our study was a nationwide study, the 93 cases originated from 55 hospitals in 14 of the 16 Federal States of Germany.

Our study has several limitations. These limitations include potential underreporting, although this might have been minimized by increased awareness during the influenza pandemic in Germany. In addition, patients with influenza could not be included when the questionnaires were not returned despite written reminders. Another limitation might be that not all children are hospitalized in pediatric hospitals. However, patients with severe cases requiring intensive care would likely have been transferred to a PICU and thus should have been captured in our study. This suggestion is supported by the fact that 11 patients had been transferred from other hospitals. An additional limitation might be that knowledge of clinical features of patients was only based on information provided in the questionnaires. Furthermore, ascertainment of underlying chronic medical conditions was not standardized and may differ from 1 physician to another. Because the survey instrument captured temporal information in days, the time from symptom onset to initiation of treatment could not be calculated in hours. Finally, even with an unexpected high number of reported severe cases, the total number of deaths in PICUs was too small to perform a multivariable analysis for factors associated with death.

This study identified a considerable number of severe cases of pandemic (H1N1) 2009 among children in Germany, confirming observations in the Americas. Our results stress the role of underlying risk factors, especially neurodevelopmental disorders, in children with severe cases of pandemic (H1N1) 2009. The results also indicate that measures that would prevent severe disease and adverse outcomes in children, including vaccination and other preventive measures, as well as early diagnosis and prompt treatment of this infection, are not used to their full extent despite availability of maximum care resources.
